# Iterative Implementation of the Dipole Interaction Model for Atomic Polarizabilities

**DOI:** 10.1002/jcc.70158

**Published:** 2025-06-27

**Authors:** Raphael F. Ligorio, Leonardo H. R. Dos Santos, Anna Krawczuk

**Affiliations:** ^1^ Institute of Inorganic Chemistry University of Goettingen Goettingen Germany; ^2^ Department of Chemistry Universidade Federal de Minas Gerais Belo Horizonte Brazil

**Keywords:** dipole moment, iterative dipole interaction model, polarizability

## Abstract

Despite its name, the dipole interaction model (DIM) serves not only to adjust dipole moments due to atomic interactions but also to assess polarizabilities. Traditionally, polarizability calculations via DIM rely on matrix inversion, posing constraints on memory usage and computational time. Recent implementations have shown significant performance boosts by employing an iterative inversion solver, albeit reducing accuracy. In this paper, we present a direct approach for computing polarizabilities via iterative cycles, eliminating the need for matrix inversion. This allows for scaling up the model to hundreds of thousands of atoms without sacrificing precision, as often happens when simplifying the standard inversion procedure to reduce computational costs. Additionally, we have addressed memory issues associated with storing extensive arrays in standard implementations. Our advancement holds promise for diverse applications, providing an efficient method for exploring polarizabilities in various systems.

## Graphical abstract

New iterative implementation of the point‐dipole interaction model, enabling rapid and precise prediction of anisotropic atomic and molecular polarizabilities.

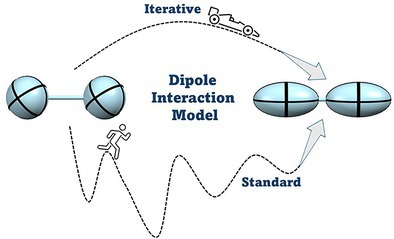

## 1 Introduction

Several electrostatic models [1, 2, 3, 4] have been devised for the investigation of interactions within molecular crystals. The dipole interaction model (DIM) derived by Applequist [4] assumes that the total electric field experienced by each atomic basin, $F_{j}^{\Omega }$ results from the cumulative effect of the external field $F_{j}^{\text{ext}}$ and the one generated by the collective dipoles, $\mu_{j}^{\Lambda }$, from adjacent sites [5, 6, 7].

(1) $$\begin{array}{c} F_{j}^{\Omega }=F_{j}^{\text{ext}}-\underset{\Lambda \neq \Omega }{\sum }T_{ij}^{\Omega \Lambda }\mu_{j}^{\Lambda } \end{array}$$

$T_{ij}^{\Omega \Lambda }$ represents here the ij component of the dipole field tensor between Ω and Λ atomic basins:

(2) $$\begin{array}{c} T^{\Omega \Lambda }=\frac{-3}{r_{\Omega \Lambda }^{5}}\left(\begin{array}{ccc} x_{\Omega \Lambda }^{2}-\frac{r_{\Omega \Lambda }^{2}}{3} & x_{\Omega \Lambda }y_{\Omega \Lambda } & x_{\Omega \Lambda }z_{\Omega \Lambda }\\ x_{\Omega \Lambda }y_{\Omega \Lambda } & y_{\Omega \Lambda }^{2}-\frac{r_{\Omega \Lambda }^{2}}{3} & y_{\Omega \Lambda }z_{\Omega \Lambda }\\ x_{\Omega \Lambda }z_{\Omega \Lambda } & y_{\Omega \Lambda }z_{\Omega \Lambda } & z_{\Omega \Lambda }^{2}-\frac{r_{\Omega \Lambda }^{2}}{3} \end{array}\right) \end{array}$$

where $x_{\Omega \Lambda }=(x_{\Omega }-x_{\Lambda })$ represents the difference in the Cartesian x coordinate between the basins Ω and Λ, and $r_{\Omega \Lambda }$ denotes the corresponding interatomic distance. Using the total electric field and the original polarizability tensors $\alpha_{ij}(\Omega )$, that is, the tensors used in the initial step of the iterative procedure that remain constant, we can recompute the dipole moments as follows:

(3) $$\begin{array}{c} \mu_{j}^{F_{i}}(\Omega )=\mu_{j}^{0}(\Omega )+\alpha_{ij}(\Omega )\left(F_{j}^{\text{ext}}-\underset{\Lambda \neq \Omega }{\sum }T_{ij}^{\Omega \Lambda }\mu_{j}^{\Lambda }\right) \end{array}$$

where $\mu_{j}^{0}(\Omega )$ denotes the permanent dipole moment component, meaning the one in the absence of any external field. In a hypothetical scenario where two particles exert mutual influence, Equation (3) serves to estimate the induced dipole moment of a particle, that is, atomic basin, Ω due to the presence of a second particle, that is, atomic basin Λ. Since the dipole moment of Λ depends on that of Ω, an iterative approach can be adopted for the computation of both dipole moments until a specified convergence threshold is met. Although noniterative methods such as variational approaches [8] exist for estimating induced dipoles, our primary aim here is to clarify the iterative cycle (IC), which is a key component in validating and understanding our proposed implementation of DIM for condensed phase polarizabilities.

To streamline the analysis, the following simplification is used: $\mu_{j}(\Omega )\equiv \mu_{\Omega }$, $T_{ij}^{\Omega \Lambda }\equiv T$, and $\alpha_{ij}(\Omega )\equiv \alpha_{\Omega }$, along with their counterparts for the particle Λ, and also for the external electric field vector, thus removing direction subscripts. At the starting of the iterative cycle, the dipole moments for both Ω and Λ particles are initialized with their respective values corresponding to no external perturbation. The superscript 0 is employed to denote the absence of iterations at this stage, meaning we start with the permanent dipole moment, as usual. The first iteration, denoted as I, is subsequently generated by directly applying Equation (3) using the initial set of dipole moments, leading to:

(4) $$\begin{array}{c} \mu_{\Omega }^{I}=\mu_{\Omega }^{0}-\alpha_{\Omega }T\mu_{\Lambda }^{0}\\ \mu_{\Lambda }^{I}=\mu_{\Lambda }^{0}-\alpha_{\Lambda }T\mu_{\Omega }^{0} \end{array}$$

In each successive iteration, the dipole moment calculated in the preceding one is utilized, while maintaining a constant polarizability. Equations (5) represent iterations II and III, respectively.

(5) $$\begin{array}{c} \begin{array}{c} \mu_{\Omega }^{II}=(1+\alpha_{\Omega }T\alpha_{\Lambda }T)\mu_{\Omega }^{0}-\alpha_{\Omega }T\mu_{\Lambda }^{0}\\ \mu_{\Omega }^{III}=(1+\alpha_{\Omega }T\alpha_{\Lambda }T)\mu_{\Omega }^{0}-(1+\alpha_{\Omega }T\alpha_{\Lambda }T)\alpha_{\Omega }T\mu_{\Lambda }^{0} \end{array} \end{array}$$

where 1 represents the 3×3 identity matrix. Each iteration is a progression from the previous one, introducing an additional term, $\alpha_{\Omega }T\alpha_{\Lambda }T$. Note that for even iterations, this product is multiplied by $\mu_{\Omega }^{0}$ term, while for odd iterations, the same product is instead multiplied by $\alpha_{\Omega }T\mu_{\Lambda }^{0}$. If infinite iterations are performed, the two parts of the summation in Equation (5) can be grouped and expanded in a power series as following:

(6) $$\begin{array}{ll} & \left(1+\alpha_{\Omega }T\alpha_{\Lambda }T+{\left(\alpha_{\Omega }T\alpha_{\Lambda }T\right)}^{2}+\text{⋯}\;)(\mu_{\Omega }^{0}-\alpha_{\Omega }T\mu_{\Lambda }^{0}\right)\\ & \;=\left[\overset{\infty }{\underset{n=0}{\sum }}{\left(\alpha_{\Omega }T\alpha_{\Lambda }T\right)}^{n}\right](\mu_{\Omega }^{0}-\alpha_{\Omega }T\mu_{\Lambda }^{0})\\ & \;={\left(1-\alpha_{\Omega }T\alpha_{\Lambda }T\right)}^{-1}(\mu_{\Omega }^{0}-\alpha_{\Omega }T\mu_{\Lambda }^{0})\; \end{array}$$

The limitation of the power series convergence becomes apparent when the modulus of at least one eigenvalue of $\alpha_{\Omega }T\alpha_{\Lambda }T$ exceeds 1. Notably, even when all eigenvalues meet the convergence criteria, Equation (6) tends to exhibit rapid escalation toward extremely high values of dipole moment as their modulus approaches 1. In such cases, despite the series convergence, it leads to unrealistic values for the newly computed dipole moments.

In a hypothetical scenario with two particles with spherical polarizabilities of 10 a.u., a common value for C, N, O atoms [9], the critical distance between the particles would be approximately 1.44 Å, falling within the typical range of covalent bond lengths for these atoms. This implies that the model should be employed cautiously for interactions within close proximity or involving highly polarizable sites, as it may yield nonrealistic results. Conversely, DIM has demonstrated high accuracy in correcting electrostatic interactions between atoms situated in different molecules [9], particularly when these atoms are separated by longer distances, typically via intermolecular contacts.

To mitigate the risk of a polarization catastrophe, meaning DIM yielding unrealistic values, Thole [10] proposed the incorporation of a damping function according to the distance between two interactive sites and their respective isotropic polarizabilities, to reduce the strength of the interaction. Among the various available models [11], we chose one particularly used in force fields like CHARMM [12], with the following form:

(7) $$\begin{array}{c} \tau_{\Omega \Lambda }=1-\left(1+\frac{s_{\Omega \Lambda }r_{\Omega \Lambda }}{2}\right)\exp(-s_{\Omega \Lambda }r_{\Omega \Lambda })\\ s_{\Omega \Lambda }=\frac{1}{b{\left(\alpha_{\Omega }\alpha_{\Lambda }\right)}^{1/6}} \end{array}$$

In this context, the tensor T is multiplied by the function $\tau_{\Omega \Lambda }$ to reduce its magnitude in short distances. The coefficient b is an arbitrary parameter tailored to replicate results obtained through ab initio quantum calculations or experiments. Here, we adopted a value of 2.6, which is sufficient to mitigate the polarization catastrophe, at least for the systems considered in this work.

When calculating polarizabilities using the DIM method, standard procedures typically rely on expensive matrix inversions rather than iterative cycles. The standard procedure involves rearranging Equation (3) to explicitly express the dipole moment and its dependence only on the external electric field. Considering the previously discussed prototypical biparticle system, the polarizabilities of the particles are then formulated as follows:

(8) μΩindμΛind=αΩ−1TTαΛ−1−1FextFext

In this expression, only the induced dipole moment (superscript ind) is considered, as the permanent one does not contribute to the net moment induced by an external field. Applequist [4] Since neither $\alpha_{\Lambda }^{-1}$ nor $\alpha_{\Omega }^{-1}$ are singular, nor are their respective Schur complements [13], for example, the complement of the 11 element, $\alpha_{\Omega }^{-1}$, is equivalent to $\alpha_{\Lambda }^{-1}-T\alpha_{\Omega }T$. Equation (8) can be then rewritten as shown in Equation (9):

(9) μΩindμΛind=(αΩ−1−TαΛT)−1−(αΩ−1−TαΛT)−1TαΛ−(αΛ−1−TαΩT)−1TαΩ(αΛ−1−TαΩT)−1FF

which leads to the effective polarizabilities, that is, the ones observed for interacting atoms, given as:

(10) αΩeffαΛeff=(αΩ−1−TαΛT)−1−(αΩ−1−TαΛT)−1TαΛ−(αΛ−1−TαΩT)−1TαΩ+(αΛ−1−TαΩT)−1=(1−αΩTαΛT)−1(αΩ−αΩTαΛ)(1−αΛTαΩT)−1(αΛ−αΛTαΩ)

The conventional method necessitates the inversion of a matrix with dimensions 3N×3N, where N denotes the number of polarizable sites. This matrix inversion involves an algorithm with a computational complexity of $O(n^{3})$ and requires the storage of a significantly large array. Consequently, it is limited to systems with a few thousand atoms. To overcome these limitations, Booth et al. [14] introduced an alternative approach that incorporates a radial cutoff for the interaction between two sites, thereby converting the matrix presented in Equation (8) into a sparse one. However, the specified threshold of 30Å may not be adequate for highly polarized sites such as sulfur atoms [9], hence necessitating larger distances and consequently increasing the computational costs. Moreover, a solver was used for inverting large sparse matrices, reducing computational costs but introducing imprecision due to the alogrithm's cost‐benefit accuracy trade‐off. In our approach, we bypass these limitations by proposing an iterative approach, similar to that used for dipole moment calculations. This eliminates the need for matrix inversion and significantly reduces RAM usage.

### 1.1 Implementing an Alternative Iterative Approach

In the preceding section, $\mu_{\Omega }$ was initially computed in the absence of an external electric field, here denoted as: $\mu_{\Omega }^{0F}=\mu_{\Omega }^{0}-\alpha_{\Omega }T{\mu_{\Lambda }}^{0F}$. In the presence of an external electric field, $F^{\text{ext}}$, the dipole moment can be determined by accounting for the fact that neighboring polarizable sites are also affected by $F^{\text{ext}}$: $\mu_{\Lambda }^{F^{\text{ext}}}=\mu_{\Lambda }^{0F}+\alpha_{\Lambda }F^{\text{ext}}$.

(11) $$\begin{array}{cc} \mu_{\Omega }^{F^{\text{ext}}} & =\mu_{\Omega }^{0}+\alpha_{\Omega }(F^{\text{ext}}-T{\mu_{\Lambda }}^{F^{\text{ext}}})\\ \mu_{\Omega }^{F^{\text{ext}}} & =\mu_{\Omega }^{0}+\alpha_{\Omega }(F^{\text{ext}}-T({\mu_{\Lambda }}^{0F}+\alpha_{\Lambda }F^{\text{ext}}))\\ \mu_{\Omega }^{F^{\text{ext}}} & =\mu_{\Omega }^{0}+\alpha_{\Omega }F^{\text{ext}}-\alpha_{\Omega }T\mu_{\Lambda }^{0F}-\alpha_{\Omega }T\alpha_{\Lambda }F^{\text{ext}} \end{array}$$

The new polarizabilities are recalculated via numerical differentiation of the dipole moments with and without the presence of f, resulting in the following expression:

(12) $$\begin{array}{c} \alpha_{\Omega }^{eff}=\frac{\mu_{\Omega }^{F^{\text{ext}}}-\mu_{\Omega }^{0F}}{\left|F^{\text{ext}}\right|}=\frac{\alpha_{\Omega }F^{\text{ext}}-\alpha_{\Omega }T\alpha_{\Lambda }F^{\text{ext}}}{\left|F^{\text{ext}}\right|}=\alpha_{\Omega }-\alpha_{\Omega }T\alpha_{\Lambda } \end{array}$$

Equation (12) is analogous to Equation (4), and the updated polarizabilities are obtained through an iterative procedure. Notably, when the effective polarizability $\alpha^{\text{eff}}$ is computed for a given particle Ω, the polarizability $\alpha_{\Omega }$ remains fixed. The newly calculated effective polarizability, $\alpha_{\Omega }^{\text{eff}}$, is then used solely to update the polarizability of another particle, Λ. The iterative scheme employed here follows the approach of Applequist [4], who modeled polarizability updates assuming point charges. This leads to an equation structurally similar to that derived from a Dyson‐like function, which represents the environment as a dipole field and solves the resulting classical electrodynamics self‐consistent screening (SCS) equation. Applying such a method typically requires the polarizability to be a continuous function; however, in practice, only discrete atomic polarizabilities are available. To address this limitation, Tkatchenko [15] later proposed adapting Dyson‐like functions for discrete models, which closely parallels the Applequist model implemented in this work. The final expression after the iterative cycle is presented in Equation (13), precisely mirroring Equation (10).

(13) $$\begin{array}{c} \alpha_{\Omega }^{eff}={\left(1-\alpha_{\Omega }T\alpha_{\Lambda }T\right)}^{-1}(\alpha_{\Omega }-\alpha_{\Omega }T\alpha_{\Lambda }) \end{array}$$

Although the proposed algorithm still has a computational complexity of $O(n^{2})$, a key advantage is in the ability to recalculate T in each IC cycle, eliminating the need to store very large arrays and thereby reducing memory‐related issues. The current implementation, for instance, requires only 1 MB of RAM for every 2000 particles. The recalculation of T in each iteration represents naturally more operations; nevertheless, it does not significantly compromise performance.

To speed up calculations, a convergence criterion was applied to each polarizable site, rather than to the entire molecule or aggregate. It means that when an atom meets this criterion, it stops updating its polarizability in the iterative cycle but still affects others. Moreover, instead of directly applying a common radial criterion to ignore long‐range interactions, a threshold based on the product Tα is used. This choice is justified by Equation (12), which shows that the polarizability of particle Ω is influenced by the sum of polarizabilities of all surrounding particles, each weighted by the respective T tensor. The maximum value of this product is determined by the highest component of α and T tensors, being the latter $2r^{-3}$. From this, it is possible to calculate the maximum distance r beyond which the interaction becomes negligible, effectively removing all interactions where the product Tα is insignificant. This approach still considers, to some extent, a radial criterion, but it is specific for each atom and scaled by its polarizability.

It is important to note that distance‐based simplifications can lead to significant deviations when dealing with thousands of interactions. Although the error of a pair of particles can be small, they can accumulate, leading to overall significant errors. Nonetheless, setting the threshold, Tα, 10,000 times lower than the IC criterion appears to provide a good balance between accuracy and efficiency. Additionally, removing a particle from the IC could accumulate errors, though does not seem to have a significant impact. Ultimately, in our implementation, the user can define the desired level of precision, with the option to retain all interactions until the total molecular or aggregate polarizability, rather than just a single atom, has converged.

### 1.2 Results

**FIGURE 1:**
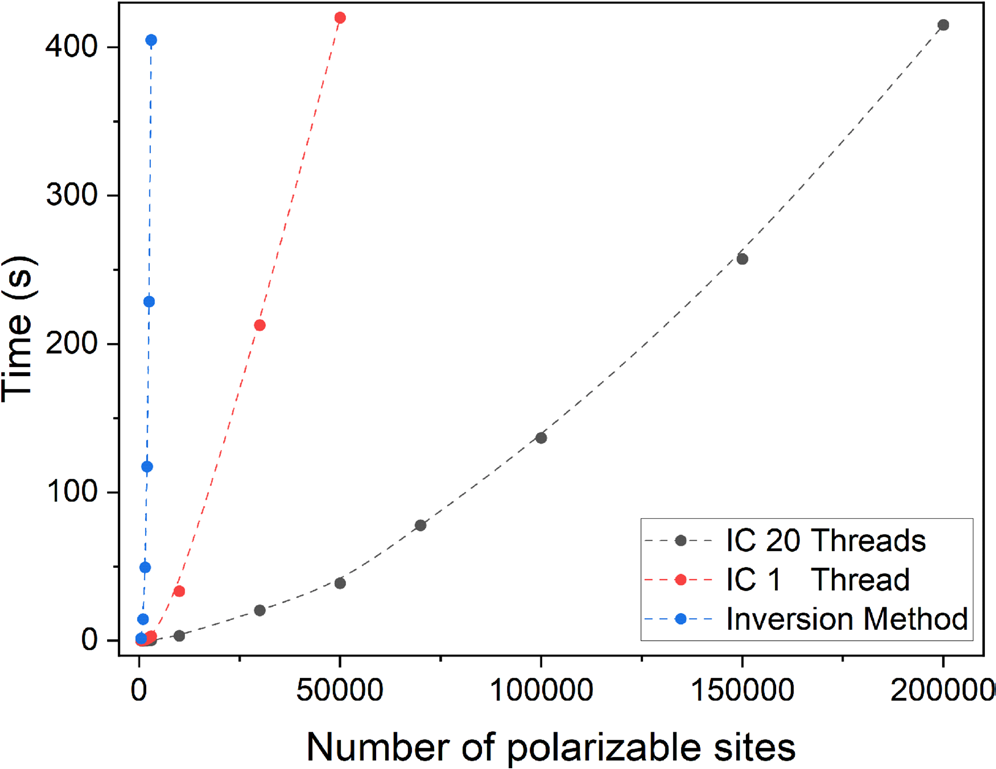
Comparison between the efficiency of inversion and IC implementations of DIM.

To demonstrate the effectiveness of our approach, we benchmarked it against the standard method by comparing the computation time for polarizabilities using the traditional inversion method, as illustrated in Figure 1 for several atomic clusters. Aggregates were constructed on a grid N×10×10, where N varied from 5 to 2000 to fit the desired number of particles with spherical polarizability of 8 a.u. Grid spacing was set to 2.5 Å. The IC convergence criteria were set to 0.001 a.u., and the new suggested cutoff was applied. Moreover, when a particle achieved convergence, it was removed from the IC. Each aggregate converged within 17 cycles without employing Thole damping factors. Calculations were performed on an Intel(R) Xeon(R) W‐1290P CPU @ 4.70GHz.

For this comparison, we employed the LU decomposition technique with LAPACK 3.8.0, followed by the matrix inversion. While more advanced optimization methods exist, such as those outlined by Booth [14], the primary objective here is to highlight the computational advantages of our approach, which eliminates the need for matrix inversion. Furthermore, the iterative cycle method is easily parallelized, as demonstrated through the use of OpenMP in Fortran 90. This parallelization significantly enhances computational efficiency, providing a notable performance boost in large‐scale calculations.

We also analyzed the errors associated with the current implementation and usual threshold cutoffs, as presented in Table 1. We considered a grid of size 500×10×10, comprising 50,000 spherical polarizable sites, each with an isotropic polarizability of 8 a.u., separated by 2.5 Å apart in all three Cartesian directions. The IC convergence was set to 0.001. The obtained results demonstrate the efficiency of the new method, reducing the errors caused by distance‐based approximations from approximately 10% to less than 0.1%.

**TABLE 1:** Comparison among different cutoff approaches: the conventional cutoff proposed by Booth [14] (30Å), current suggested approach ($T\alpha <10^{-7}$), and a model without radial criteria to discard interactions (reference).

	$\alpha_{XX}(a.u.)$	$\alpha_{YY}(a.u.)$	1st iter. time (s)	Total time (s)
No cutoff	584368.7	350807.1	4.9	83.3
Usual cutoff 30Å	517900.2 (−11.37)	364492.7 (3.90)	1.0	16.5
Newcutoff $10^{-7}$	583780.7 (−0.10)	350913.0 (0.03)	2.8	38.7

*Note:* All three cases achieved convergence within 17 cycles. Notably, in the reference model, all atoms remained in the IC even upon meeting the convergence criteria of 0.001 a.u. Values in parentheses indicate the percentual deviation relative to the reference.

Moreover, Table 2 presents the polarizability results for the 3‐hydroxypyridine molecule calculated using both approaches. The initial isotropic polarizabilities, obtained from Litman [11], were 2.914, 13.932, 11.485, and 5.616 a.u. for the H, C, N, O atoms, respectively. The results indicate that both methods yield closely matching values, with only minor numerical deviations. As Supporting Information, we include our newly implemented model along with the inversion approach. Additionally, a brief guide is provided to assist with using the code.

**TABLE 2:** Coordinates and polarizabilities obtained using both approaches for the 3‐hydroxypyridine. Coordinates are given in Å, whereas polarizabilities in a.u.

Iterative cycles	x	y	z	$\alpha_{xx}$	$\alpha_{yy}$	$\alpha_{zz}$	$\alpha_{xy}$	$\alpha_{xz}$	$\alpha_{yz}$
C	−2.46746	0.66441	0.04278	21.27583	23.37294	8.97355	0.32135	−0.64606	−0.27980
C	−2.65776	−0.71868	0.08042	21.54539	23.83767	8.89901	−1.01159	−0.62730	−0.24310
C	−1.16902	1.17399	−0.03447	21.22266	23.03737	9.13516	−0.10575	−0.62315	−0.35752
N	−0.10589	0.32665	−0.07227	13.66287	19.24732	7.47641	−2.45922	−0.26799	−0.10646
C	−1.55379	−1.57553	0.04069	21.59685	22.85542	8.73038	−0.52538	−0.65457	−0.21489
C	−0.26693	−1.02359	−0.03680	22.08649	21.84745	9.04607	0.89087	−0.69669	−0.18486
O	−1.73841	−2.91046	0.07707	4.14518	12.26983	3.78817	−0.79109	−0.00424	−0.16335
H	−0.99729	−3.53242	0.05133	2.54240	5.16423	2.06688	−2.24484	0.02005	−0.00693
H	0.60395	−1.66516	−0.06883	5.53047	3.21068	1.75415	−2.75480	−0.14191	0.12205
H	−1.00634	2.24307	−0.06437	1.45248	7.54674	1.78357	1.08640	−0.00454	−0.17797
H	−3.31692	1.33442	0.07313	4.89825	3.68209	1.76635	−3.21561	−0.09871	0.11623
H	−3.66034	−1.12314	0.14029	6.36461	2.04139	1.76340	2.02679	−0.27647	−0.11098

Inverting	x	y	z	$\alpha_{xx}$	$\alpha_{yy}$	$\alpha_{zz}$	$\alpha_{xy}$	$\alpha_{xz}$	$\alpha_{yz}$
C	−2.46746	0.66441	0.04278	21.27584	23.37294	8.97355	0.32136	−0.64606	−0.27980
C	−2.65776	−0.71868	0.08042	21.54541	23.83768	8.89901	−1.01160	−0.62730	−0.24310
C	−1.16902	1.17399	−0.03447	21.22267	23.03735	9.13515	−0.10573	−0.62316	−0.35752
N	−0.10589	0.32665	−0.07227	13.66288	19.24729	7.47642	−2.45921	−0.26799	−0.10646
C	−1.55379	−1.57553	0.04069	21.59689	22.85540	8.73037	−0.52540	−0.65458	−0.21489
C	−0.26693	−1.02359	−0.03680	22.08653	21.84742	9.04607	0.89085	−0.69669	−0.18486
O	−1.73841	−2.91046	0.07707	4.14518	12.26982	3.78817	−0.79108	−0.00424	−0.16335
H	−0.99729	−3.53242	0.05133	2.54240	5.16423	2.06689	−2.24483	0.02004	−0.00693
H	0.60395	−1.66516	−0.06883	5.53048	3.21068	1.75415	−2.75480	−0.14191	0.12205
H	−1.00634	2.24307	−0.06437	1.45248	7.54674	1.78357	1.08640	−0.00454	−0.17797
H	−3.31692	1.33442	0.07313	4.89825	3.68209	1.76635	−3.21561	−0.09871	0.11623
H	−3.66034	−1.12314	0.14029	6.36461	2.04139	1.76340	2.02679	−0.27647	−0.11098

In a nutshell, this paper presents a novel method for calculating atomic polarizabilities using the DIM with an iterative procedure. Our approach matches the performance of previous methods while avoiding the costly large matrix inversions they rely on. While applying DIM to large systems often involves approximations that may impact accuracy, our method improves efficiency without them. Moreover, a larger molecule, $C_{50}H_{152}$, was employed to demonstrate the accuracy of our method for extended systems, showing deviations of 0.0001 atomic units or less per atomic and molecular component when compared to the conventional inversion method. The numerical values are provided in the Supporting Information file. It also overcomes memory constraints, resulting in a more practical and scalable solution. These enhancements make the approach more suitable for larger systems and broader applications. This method was successfully implement in our software GruPol [16], aiming at the prediction of solvent effects on proteins electrostatic properties of proteins.

## Supporting information




**Data S1.** Supporting Information.

## Data Availability

The data that support the findings of this study are openly available in GRO.data at https://doi.org/10.25625/TSH366.
