# Supplementary material for: Iterative Implementation of the Dipole Interaction Model for Atomic Polarizabilities
Source: J Comput Chem. 2025 Jun 27;46(17):e70158. doi: 10.1002/jcc.70158 (PMC12204055; doi:10.1002/jcc.70158)
Supplement: Supplementary file 1 — Data S1. Supporting Information. [file JCC-46-0-s001.pdf]

Supporting Information for:
Iterative Implementation of the Dipole Interaction Model for Atomic Polarizabilities

Raphael F. Ligorio, Leonardo H. R. Dos Santos, Anna Krawczuk

1 How to use the routines

Here we aim to demonstrate the process of creating an input file for running the program utilized to calculated polarizabilities in condensed phase via DIM. Both versions, inversion, and SCF are available. To execute the program, the user needs to ensure that all Fortran libraries are placed in the same directory as the executable cryspol-test.exe. Before running the software, the user must prepare two files. The first one should contain the path where the input file is located. This file should be named pathout.txt and placed alongside the executable, as illustrated below. It is worth noting that paths with spaces in their names must be avoided.

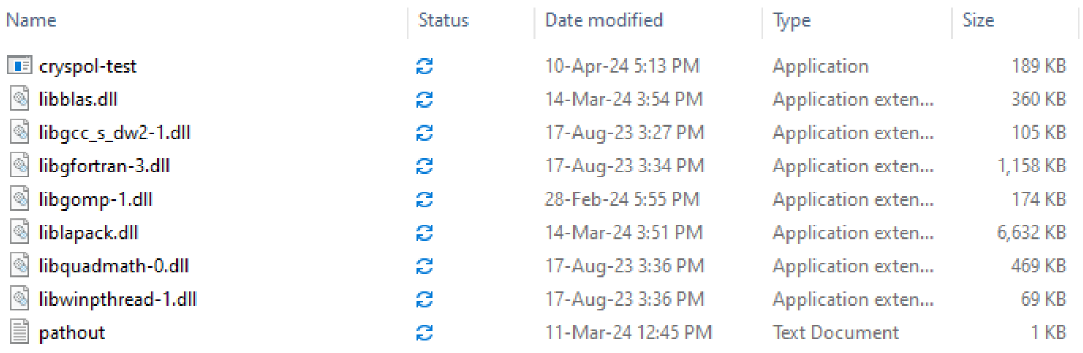

Figure 1: Screenshot of the folder containing the files required to run Cryspol-test.exe

Double clicking with the left mouse button on cryspol-test.exe will initiate the program, which will open pathout.txt and locate your input, the second file that must be prepared. This file must be named unitcel.dat and has the following structure:

....

(L1) | 10.0 10.0 10.0 90.0 90.0 90.0
(L2) | covalent 1
(L3) | thole 2.6
(L4) | 1
(L5) | 1 0 0, 0 1 0, 0 0 1, 0 0 0
(L6) | 3 3 3 0 50
(L7) | 2
(L8) | C 1 1, 0.0 0.0 0.0, 0.0 0.0 0.0, 8.0 8.0 8.0 0.0 0.0 0.0
(L9) | O 1 2, 3.0 0.0 0.0, 0.0 0.0 0.0, 8.0 8.0 8.0 0.0 0.0 0.0

....

- **L1: Cell lattice parameters** The first line contains the cell lattice parameters, A, B, C, α, β, γ. These values must be provided in Angstroms and Degrees, respectively.
- **L2: Covalent interaction** This line includes a keyword, 'covalent', which specifies whether atoms within the same molecule interact with each other (0 to disable, 1 to enable).

- **L3: Thole damping** This line contains the 'thole' keyword, which is applied to damp the interactions between two polarizable sites. This variable is set to 0 when no damping is applied, or to a value greater than 0 to increase the strength of the damping.
- **L4: Number of symmetry elements** The number 1 in this line refers to the number of symmetry elements applied to create a super-cell, based on a given space group, and it must be adapted to each particular case.
- **L5: Symmetry element** This line contains the symmetry elements. In this instance, there is only 1 element represented by the identity matrix, described sequentially by the rows of the matrix. The last three numbers refer to the translational part of the symmetry element along A, B, and C, respectively.
- **L6: Replications and cutoff** This line specifies how many replications of the cell must be created in each direction, A, B, and C, respectively (3 repetitions along each direction). Additionally, it is necessary to include a cutoff factor if a spherical aggregate is desired. The program identifies the central molecule and removes all molecules outside the specified radius of the sphere. This cutoff value can be 0 if no cutoff is applied or greater than 0. Notably, this cutoff is given in Bohrs. Finally, the last number in this line is the number of convergence cycles in case of SCF calculations.
- **L7: Number of atoms in the molecule** This line contains the number of atoms in the molecule.
- **L8: Atom parameters** This line contains a list of parameters for each atom. Firstly, the atom label is followed by the number of the molecule in which this atom is located (usually, always 1). Next, the atom number ranges from 1 to the total number of atoms in the molecule. The following three values represent the Cartesian coordinates of the atoms (in Bohrs), x, y, z, respectively. The subsequent three numbers are for atomic dipole moments, which have been removed from this version. Therefore, these lines must be 0 (or any other number) and have no impact here. The sequence of these last 6 numbers represents the atomic polarizabilities α_{xx}, α_{yy}, α_{zz}, α_{xy}, α_{xz}, α_{yz}, respectively, given in atomic units ($Bohr^3$).

It is important to note that commas in the input file are optional and can be removed. Moreover, if the user wants to calculate a single molecule without crystalline packing, they should always enable the covalent option, provide only the identity symmetry element (which must be included in all calculations), set A, B, C high enough to encompass the molecule, and set the number of replications in each direction to 1. Then, they should add each atom of the desired molecule. Upon program execution, the user is asked to specify convergence criteria and the number of threads for parallel calculations.

2 Polarizabilities for the $C_{50}H_{102}$ molecule

Table 1: Input coordinates (in a.u.) for polarizability computations of the $C_{50}H_{102}$ molecule.

Atom type	x	y	z
C	-14.75370793	0.48724798	-0.21571268
C	-11.87521450	0.95786634	-0.72085641
C	-15.85687561	1.22898591	2.39940908
C	-10.12596703	-0.99390349	0.62138102
C	-11.22558202	3.75374077	-0.29925764
C	-7.21646313	-0.84743052	0.14749343
C	-5.82341087	-3.40527456	0.69725368
C	-6.01525625	1.27781653	1.85150076
C	-3.10546890	1.33983747	1.96955220
C	-2.14100740	3.49154074	3.67924762
C	0.70810073	3.30578028	4.30474825
C	1.27486855	1.08292867	6.11735837
C	4.06237148	0.45177775	6.83985884
C	3.95681116	-2.14677107	8.25983975
C	5.45387416	2.48195248	8.42755329
C	6.46093018	-3.44125502	9.02550166
C	7.50135882	-2.70906024	11.63451959
C	7.60521839	1.24691944	-0.67259270
C	10.35658968	0.90383896	0.26049928
C	7.15814606	3.76422878	-2.01800487
C	11.53852130	2.98726629	2.08939887
C	10.63373748	-1.80726218	1.31440170
C	14.47646564	2.45462699	2.18670107
C	10.20654511	3.17000318	4.80178503
C	16.26766512	4.77173833	1.82689648
C	7.69373334	4.73027765	4.68125805
C	11.83819469	4.27010950	6.95910080
C	6.18289420	5.00549793	7.16838840
C	19.07208113	3.94422556	1.61845926
C	16.01691684	6.90136955	3.76923656
C	-6.50671834	-5.84708918	-0.76261944
C	-8.79854509	-7.50850200	-0.01668632
C	-8.73164864	-8.79971672	2.59900236
C	-9.25187242	-7.09179764	4.87034444
C	-16.01566962	-5.64292275	1.85970219
C	-16.70135815	-5.31191765	4.63601792
C	-18.86636512	-7.18416764	5.28563150
C	-17.41711229	-2.55062527	5.31679315
C	-19.54063968	-7.29872308	8.11193059
C	-21.56739400	-9.28962922	8.61503341
C	20.03548438	2.32878987	3.83961011
C	22.91639666	2.14363412	3.75396754
C	23.96508343	0.38877415	5.81468031
C	26.80088785	0.86952146	6.09572096
C	28.06129997	-0.79325196	8.07215177
C	30.87928424	-0.18182982	8.58441978
C	31.44901894	2.31387990	10.02424286
C	-15.49089160	-0.37943889	4.83447736
C	32.39301467	4.65633251	8.53235772
C	30.54143831	5.84686241	6.65449522
H	-15.35180747	-1.41204405	-0.81475709
H	-15.73145423	1.76340155	-1.57289787
H	-11.62025213	0.63782167	-2.78663365

H	-10.78423557	-2.75870344	-0.20558373
H	-10.45547925	-1.07640911	2.67827653
H	-6.88160297	-0.45073840	-1.88954103
H	-6.63425621	3.17055120	1.28314557
H	-6.66825246	0.97332433	3.82727017
H	-12.64180237	4.97907950	-1.24858241
H	-9.42037964	4.22921574	-1.23716844
H	-11.16040523	4.23533847	1.74064920
H	-2.26837520	1.53629380	0.05276126
H	-2.51129999	-0.46827509	2.79474059
H	-2.52980045	5.30723112	2.69433924
H	-3.19468305	3.51263012	5.49845289
H	1.81979109	3.15010432	2.53036737
H	1.16704056	5.09178300	5.25530134
H	0.57576294	-0.63661224	5.17081151
H	0.15599721	1.34715072	7.87752848
H	5.15648735	0.11138069	5.08583035
H	3.08566453	-3.48364167	6.88800916
H	2.67886235	-2.07290153	9.92754538
H	7.89476282	-3.23916730	7.50625322
H	6.03604328	-5.49231127	9.21524945
H	8.11782654	-0.73393334	11.83411287
H	9.17526276	-3.90389872	12.05129954
H	6.05206820	-3.08521099	13.10552321
H	6.22541313	0.94180364	0.85761617
H	7.19964453	-0.26563935	-2.07584951
H	7.43895994	5.39236345	-0.73446246
H	5.18454984	3.82708120	-2.72863784
H	8.46309911	3.95220022	-3.65099615
H	11.48356796	0.97691482	-1.51529890
H	11.36810545	4.83016878	1.10288422
H	9.43271958	-2.11077172	3.00189280
H	12.62073188	-2.24932672	1.81370616
H	10.04531335	-3.20980089	-0.13207323
H	15.01636150	1.29971850	0.51782381
H	14.93589680	1.23200947	3.82059942
H	9.84016426	1.21184605	5.42947775
H	13.66218098	3.27110373	7.16436327
H	10.91676234	3.99141212	8.81568494
H	12.10045142	6.32859239	6.77853710
H	6.37591123	3.94995144	3.30691412
H	8.15856912	6.66904614	4.01760377
H	15.78986578	5.66644989	-0.01691308
H	14.10844072	7.74641901	3.63510353
H	17.34636079	8.45935745	3.30946525
H	16.45512633	6.25920150	5.71187900
H	-5.67884652	-3.76239574	2.73022521
H	-3.85920661	-3.07570565	0.03647179
H	-4.85042668	-7.13078277	-0.56572847
H	-6.65150945	-5.35366178	-2.79984287
H	-8.77146525	-9.08472580	-1.40813231
H	-10.64479240	-6.60235757	-0.29880411
H	-10.28608385	-10.21416073	2.58567976
H	-6.92686201	-9.83848240	2.87108569
H	-11.00353874	-5.99964709	4.56258301
H	-7.70529849	-5.78230924	5.30698545
H	-9.48340213	-8.28249762	6.58211856
H	-14.29807513	-4.54357233	1.46618482
H	-15.53535695	-7.64641450	1.45297560
H	-17.60234362	-5.05710645	0.61614647

H	-15.02567787	-5.85971258	5.77066850
H	-18.25222177	-9.11482920	4.71878809
H	-20.58320372	-6.69389609	4.17484821
H	-17.82552073	-7.78806866	9.22210917
H	-20.28517440	-5.45612294	8.77664311
H	-20.89192891	-11.18897795	8.02940608
H	-23.32242405	-8.82609735	7.56039399
H	-22.01068687	-9.34067083	10.66574950
H	19.35979252	2.83408477	-0.14271241
H	20.23356588	5.68801171	1.43275549
H	19.17864300	0.41511699	3.74011582
H	19.52474705	3.20793006	5.67467022
H	23.58651382	1.46168726	1.88167975
H	23.64176952	4.09753935	4.04600642
H	23.55049556	-1.61035232	5.31779471
H	23.06271846	0.82696475	7.66315751
H	27.78596631	0.62844861	4.25425466
H	26.90338681	2.82053541	6.75686190
H	27.92969917	-2.78085108	7.39901105
H	27.01053450	-0.65828745	9.88759648
H	32.10976304	-0.39947002	6.89707986
H	31.42896891	-1.71818031	9.91289997
H	33.09652335	1.87871287	11.25870898
H	29.86574646	2.78502738	11.32148581
H	34.15943978	4.15842645	7.50931459
H	32.87332734	6.08914470	9.98569237
H	-19.28367420	-2.02318273	4.51112566
H	-17.65077740	-2.58866554	7.40148659
H	-17.93281960	1.46038334	2.16789826
H	-15.14809458	3.15921282	2.80568213
H	-13.52757551	-0.88053859	5.33744790
H	-16.04601868	1.02236283	6.30574555
H	30.05524858	4.49075437	5.23564814
H	31.40536618	7.45392660	5.78298954
H	28.86980205	6.42785990	7.63254388
H	7.29069172	1.67513226	8.91910986
H	4.39609780	2.85349230	10.20306801
H	4.52191602	6.20084664	6.82082926
H	7.30216238	6.11463716	8.55306916

Table 2: Atomic polarizabilities (in a.u.) of the $C_{50}H_{102}$ molecule calculated using the iterative method with a convergence threshold of 10^{-12}. A damping factor of 0.39 was employed. Initial spherical atomic polarizability tensors were employed, with values of 9.051 a.u. for C-atoms and 3.240 a.u. for H-atoms.

Atom	α_{xx}	α_{yy}	α_{zz}	α_{xy}	α_{xz}	α_{yz}
1	12.78142	9.71352	9.56556	0.47326	-0.68739	-0.85339
2	13.01017	10.62960	8.26840	-0.12353	-0.08802	-1.07830
3	10.96374	9.86144	10.13911	0.03116	-1.27468	-1.20170
4	14.65020	10.44611	7.60682	-0.13228	0.55754	-0.79169
5	11.90004	11.51978	8.46756	0.20397	0.21278	-1.06776
6	13.97291	10.54449	7.86536	0.35079	0.67512	0.69785
7	11.60858	12.23668	7.88682	1.02017	0.48389	0.92516
8	15.26382	10.08871	7.49593	1.89534	1.99596	0.64908
9	15.37230	9.12489	8.31017	2.23092	1.98363	0.80703
10	15.18539	9.41032	8.55389	0.96619	2.14239	0.56772
11	15.31749	8.78849	8.84054	0.73934	1.78383	-0.64944
12	14.41028	8.46730	8.86412	-0.12919	1.94025	-1.19341
13	14.02577	9.78994	9.04063	1.06119	1.16471	-1.36381
14	11.42429	10.23042	9.45539	-0.33019	1.09828	-2.11407
15	12.17685	10.13718	9.52669	1.19610	-0.27627	-0.60076
16	11.03203	9.95936	10.76960	-0.19090	0.99122	-1.50245
17	10.15255	9.69651	11.45282	-0.07556	1.09813	-1.43337
18	12.53697	8.93951	10.68516	0.08278	0.61722	-0.63741
19	12.51919	9.21292	9.41119	0.50419	1.16800	0.47866
20	11.16672	8.94325	11.18891	-0.18472	0.67648	-1.11403
21	14.40340	8.12409	9.65006	1.18158	-0.17525	0.90842
22	11.61983	10.70218	9.33129	0.22132	-0.18999	0.29784
23	15.78518	8.54313	7.98928	1.56346	-0.08257	0.17435
24	13.56262	8.04598	9.93242	0.60750	-0.63397	0.76855
25	14.95171	9.51868	8.19052	0.56356	0.19881	0.11793
26	13.89186	8.42496	9.81893	0.58506	-0.96043	0.21665
27	13.56218	8.42171	10.03894	1.38037	-0.06718	0.65329
28	12.71692	10.05029	9.71605	0.88606	-0.96837	-0.11662
29	15.20605	8.48971	8.87673	-0.55290	0.44114	-0.71497
30	12.61510	10.38894	8.49404	1.10027	-0.26477	0.63732
31	10.81797	11.83651	8.87010	1.41190	-0.60990	0.62723
32	11.19196	11.05607	9.53260	0.81531	-0.85691	0.15449
33	9.94822	10.64620	9.70530	-0.14447	-0.53678	-0.23346
34	11.67834	10.34931	10.05492	0.27169	-0.45136	-0.54391
35	12.15855	10.11683	10.22523	1.27292	-1.15311	0.01037
36	11.28442	10.88267	9.46154	1.38332	-2.09658	-0.84320
37	11.35513	10.13400	10.42272	2.31936	-2.05386	-1.43480
38	10.45697	11.89255	8.84028	1.21151	-1.33579	-0.91790
39	11.10715	10.17230	10.55795	2.06719	-1.93440	-1.44659
40	11.87376	10.74258	10.13130	2.08776	-1.75063	-1.46559
41	16.08700	8.14048	8.19001	-1.01835	1.97880	-0.87757
42	15.85195	8.25830	9.05532	-1.19271	2.02837	-0.92166
43	14.92293	8.78027	8.90675	-0.24409	2.52507	-0.83322
44	14.79300	9.06898	9.17687	-0.41423	2.56578	-0.52547
45	13.08551	9.70020	9.35474	0.11214	2.10546	-0.61260
46	12.58886	10.45942	9.14722	0.38235	1.46807	0.28271
47	10.80799	10.86063	9.53622	1.08344	1.00984	-0.04148
48	11.26424	11.11201	9.51778	1.08096	-1.45236	-1.11413
49	11.23630	10.40252	9.69909	0.67568	1.06591	-0.68752
50	11.89279	11.11738	9.64661	0.88393	1.02761	-0.77865
51	2.90011	4.53233	2.47293	0.93036	0.18459	0.12132
52	3.46137	3.03164	3.52278	-0.89711	1.05479	-1.26525
53	2.68894	2.15912	4.62360	-0.06347	-0.11285	0.11706

54	3.69069	4.90487	1.89535	0.63035	0.10273	0.41892
55	3.80600	2.32304	3.73813	0.15020	-0.59324	-0.31272
56	2.95466	2.35654	4.16510	0.08695	-0.28132	-0.42227
57	4.04613	4.29982	1.79833	-0.49438	0.74580	-0.54877
58	3.78340	2.12685	3.52777	0.56938	-0.23585	0.11039
59	3.82312	3.96707	2.56229	-1.19990	0.82769	-1.19803
60	5.13739	2.84152	2.46214	0.70654	-0.68337	-0.74531
61	2.97482	2.79389	3.81325	0.13856	0.05004	0.64533
62	3.80807	2.00349	3.79651	0.64588	-0.51363	-0.04268
63	4.17654	3.84107	2.14555	0.12757	1.12691	-0.46193
64	3.24712	4.32440	2.26954	-0.19231	0.55788	-0.93976
65	4.09183	1.98676	3.54286	0.05764	-0.55016	0.39815
66	4.79162	1.68204	3.57769	0.29904	-0.74435	-0.08048
67	3.88409	3.99269	2.24445	0.53434	0.65736	0.93993
68	4.26337	3.46235	2.43381	0.71738	1.18363	0.51188
69	4.05280	1.86132	3.61609	-0.08986	-0.70780	-0.04661
70	4.15522	2.20716	3.86380	0.30880	-1.11618	0.00008
71	3.40444	3.44017	2.81758	0.64822	1.00759	0.61705
72	3.73273	2.10323	3.79665	0.16166	-1.04833	-0.50994
73	3.82067	2.41615	3.76679	0.31850	-1.47655	-0.54030
74	2.36008	5.25647	2.18406	0.42441	0.12709	-0.83947
75	2.50717	4.48293	2.74806	0.77717	0.34777	-0.46404
76	4.18098	3.06171	2.73724	-1.44022	1.19324	-0.95301
77	3.20499	2.14665	4.41855	0.45272	-1.20715	-0.70222
78	4.52802	1.93901	4.00537	0.42281	-1.51278	-0.17873
79	2.75049	3.33620	3.62506	0.48568	0.70473	1.49432
80	2.58673	3.46322	3.49744	0.04362	0.40527	0.91997
81	5.41731	1.94482	2.95596	-0.22838	1.33684	-0.38828
82	3.24537	2.03942	4.60559	0.26926	-1.22900	-0.71416
83	3.76394	1.92902	4.09884	0.18689	-0.89200	0.11619
84	3.55976	3.50537	2.66738	0.16379	0.30460	-0.90419
85	3.53729	2.57726	3.89104	0.27680	-1.44388	-0.45743
86	5.43264	2.78593	1.87988	-0.41669	0.51387	0.00577
87	2.47291	4.05117	3.22936	0.47405	0.47692	1.55044
88	4.03936	2.71702	3.14472	0.10711	-0.36384	1.27905
89	4.09571	2.63537	3.11771	-0.10911	0.43902	-0.95045
90	3.24767	3.97477	2.65883	0.62024	-0.50915	-0.49489
91	5.68672	2.04546	2.50912	-0.39746	0.00846	-0.08070
92	3.86186	1.84643	4.56911	0.49527	-0.80665	-0.27132
93	3.28588	4.31609	2.10393	0.78093	-0.17810	0.21080
94	4.60153	2.10993	3.79573	0.79350	0.67575	0.63341
95	3.02396	4.30165	2.39258	0.36712	-0.27794	-0.77892
96	3.14735	2.53121	3.99151	0.02682	0.37957	-1.13923
97	5.53189	3.00477	1.86486	-0.31696	-0.16882	0.02181
98	3.98813	4.45608	1.90840	1.52684	-0.27219	-0.27152
99	3.43244	2.28770	3.94404	0.06109	0.34900	-0.23436
100	2.91127	2.85033	3.85498	0.57196	0.35266	-0.44461
101	5.77718	2.81041	1.90888	1.07486	-0.35657	0.36302
102	4.28605	3.50549	1.95210	-1.22372	-0.25029	0.23105
103	2.25268	2.48722	4.70959	0.07136	-0.07349	-0.14898
104	2.20464	4.18112	3.52800	0.25012	-0.02038	1.63565
105	5.00849	3.11581	2.08661	-0.99259	-0.04780	0.08494
106	3.71386	4.08162	2.04626	1.43772	-0.21795	-0.06478
107	4.21136	3.46368	2.07800	-1.43563	0.18869	-0.28544
108	5.31075	2.89492	2.03585	-1.27451	0.05118	-0.36318
109	4.26572	3.38540	2.45099	1.64598	0.22431	-0.03959
110	2.42349	3.12007	4.31249	0.17070	-0.29959	-1.51148
111	5.50501	3.53656	2.14035	1.52884	-0.99838	-0.54579
112	2.95608	4.56598	2.34611	-0.36296	-0.44443	0.75143
113	3.96452	2.47012	3.36494	-0.02506	1.05781	-0.40707

114	4.70224	2.36979	2.63546	-0.32718	0.39445	-0.54590
115	2.63642	4.54186	2.46313	-0.06677	-0.64913	0.38227
116	4.43615	2.27559	2.93894	0.25172	0.74692	-0.48829
117	4.01528	2.26826	3.22633	-0.00765	0.73007	-0.69156
118	2.75144	4.24507	2.72199	-0.28818	-0.89204	0.43005
119	2.53996	4.92256	2.28497	-0.24150	-0.39427	0.19324
120	5.01209	2.25516	2.59090	0.20448	0.60832	-0.34538
121	2.76033	2.29116	5.22356	0.44707	-1.04930	-0.45532
122	3.31885	2.27941	4.05980	-0.27816	-0.34134	0.91264
123	4.37446	3.63752	2.04554	1.01846	-0.15882	-0.57842
124	4.43553	3.74507	1.74368	0.62318	0.48409	-0.09722
125	4.02252	2.12811	3.48001	-0.59916	-0.01842	0.50474
126	3.58155	2.00711	4.10457	-0.46902	-0.41292	0.51233
127	4.05064	3.83705	1.92405	0.64652	0.68063	0.02559
128	3.19505	4.45744	2.05296	0.41676	0.55669	0.35110
129	3.93631	1.82813	3.76813	-0.30153	-0.38348	0.03735
130	3.76724	2.04429	3.92892	-0.10848	-0.50424	0.19312
131	3.67142	4.40879	2.03000	0.59335	0.77954	0.60279
132	2.72865	4.93184	2.17890	0.08613	0.40455	0.56366
133	3.47449	2.02872	4.01118	0.11412	-0.67219	-0.21367
134	3.76105	2.44936	3.45981	-0.29959	-1.02106	0.41921
135	2.93394	4.01373	3.12491	-0.71689	0.84541	-1.38619
136	4.35727	2.39996	3.44791	-0.22080	1.72751	-0.42252
137	3.77776	2.35211	3.43616	-0.12720	-0.81991	0.46235
138	4.93220	2.31756	2.52589	-0.15465	-0.86533	0.05664
139	2.69906	3.73194	3.47488	0.77149	0.74851	1.31573
140	4.72257	2.76230	2.16219	-0.41634	0.32586	-0.49036
141	2.38098	2.72466	4.68854	0.49180	-0.80126	-0.12396
142	5.47582	2.42187	2.13030	-0.58383	-0.04978	-0.34453
143	2.89235	4.68664	2.18157	0.67351	-0.36746	0.09247
144	5.39892	2.36367	2.31049	-0.27845	-0.13464	-0.15905
145	2.37584	3.67777	3.63593	-0.24589	-0.64153	1.23186
146	3.09823	3.53003	3.50126	0.95955	0.74267	0.95612
147	3.00290	4.62053	2.67653	1.27418	-0.40728	-1.24531
148	4.72897	2.77053	2.36948	-0.46495	-0.55778	0.38198
149	5.08043	2.87139	2.56404	-0.37493	0.43213	-0.86900
150	3.16532	2.23884	4.42766	-0.03976	-1.07699	0.45590
151	5.07799	3.29602	2.00024	-0.92511	0.25312	0.06239
152	3.58402	3.18080	3.30336	0.92472	0.56586	1.27474

Table 3: Atomic polarizabilities (in a.u.) of the $C_{50}H_{102}$ molecule calculated using the standard inversion method. A damping factor of 0.39 was employed. Initial spherical atomic polarizability tensors were employed, with values of 9.051 a.u. for C-atoms and 3.240 a.u. for H-atoms.

Atom	α_{xx}	α_{yy}	α_{zz}	α_{xy}	α_{xz}	α_{yz}
1	12.78140	9.71365	9.56549	0.47328	-0.68736	-0.85343
2	13.01011	10.62958	8.26846	-0.12352	-0.08799	-1.07832
3	10.96385	9.86140	10.13905	0.03113	-1.27466	-1.20169
4	14.65021	10.44604	7.60685	-0.13229	0.55754	-0.79171
5	11.90016	11.51977	8.46751	0.20391	0.21282	-1.06777
6	13.97294	10.54448	7.86529	0.35075	0.67510	0.69781
7	11.60852	12.23668	7.88689	1.02015	0.48390	0.92514
8	15.26374	10.08870	7.49597	1.89528	1.99592	0.64900
9	15.37218	9.12497	8.31016	2.23086	1.98358	0.80694
10	15.18535	9.41037	8.55386	0.96618	2.14232	0.56767
11	15.31751	8.78844	8.84055	0.73934	1.78377	-0.64945
12	14.41026	8.46728	8.86417	-0.12919	1.94027	-1.19337
13	14.02580	9.78984	9.04065	1.06118	1.16475	-1.36381
14	11.42428	10.23035	9.45542	-0.33018	1.09828	-2.11408
15	12.17688	10.13713	9.52665	1.19605	-0.27621	-0.60076
16	11.03199	9.95932	10.76970	-0.19087	0.99123	-1.50236
17	10.15253	9.69641	11.45296	-0.07556	1.09814	-1.43330
18	12.53696	8.93945	10.68517	0.08277	0.61714	-0.63737
19	12.51911	9.21300	9.41118	0.50420	1.16794	0.47866
20	11.16673	8.94326	11.18891	-0.18473	0.67656	-1.11391
21	14.40332	8.12414	9.65007	1.18159	-0.17527	0.90838
22	11.61981	10.70223	9.33120	0.22134	-0.19002	0.29779
23	15.78510	8.54321	7.98927	1.56352	-0.08258	0.17428
24	13.56258	8.04600	9.93242	0.60749	-0.63402	0.76850
25	14.95168	9.51877	8.19049	0.56361	0.19877	0.11787
26	13.89182	8.42499	9.81890	0.58507	-0.96043	0.21668
27	13.56214	8.42164	10.03895	1.38035	-0.06720	0.65327
28	12.71695	10.05028	9.71600	0.88601	-0.96833	-0.11659
29	15.20598	8.48971	8.87680	-0.55290	0.44116	-0.71491
30	12.61509	10.38896	8.49400	1.10028	-0.26474	0.63733
31	10.81792	11.83653	8.87008	1.41188	-0.60992	0.62727
32	11.19183	11.05602	9.53265	0.81530	-0.85688	0.15446
33	9.94818	10.64620	9.70537	-0.14444	-0.53680	-0.23340
34	11.67827	10.34934	10.05503	0.27179	-0.45140	-0.54392
35	12.15852	10.11686	10.22526	1.27292	-1.15314	0.01035
36	11.28448	10.88268	9.46154	1.38336	-2.09661	-0.84320
37	11.35513	10.13396	10.42279	2.31942	-2.05393	-1.43479
38	10.45685	11.89256	8.84032	1.21148	-1.33577	-0.91787
39	11.10720	10.17232	10.55801	2.06724	-1.93442	-1.44661
40	11.87391	10.74257	10.13121	2.08784	-1.75065	-1.46566
41	16.08690	8.14048	8.19006	-1.01839	1.97882	-0.87758
42	15.85191	8.25819	9.05540	-1.19275	2.02838	-0.92170
43	14.92289	8.78027	8.90680	-0.24406	2.52506	-0.83326
44	14.79299	9.06895	9.17688	-0.41419	2.56576	-0.52542
45	13.08555	9.70015	9.35468	0.11211	2.10540	-0.61262
46	12.58887	10.45937	9.14726	0.38232	1.46796	0.28267
47	10.80793	10.86063	9.53625	1.08342	1.00983	-0.04153
48	11.26427	11.11197	9.51777	1.08091	-1.45236	-1.11410
49	11.23633	10.40248	9.69910	0.67563	1.06597	-0.68762
50	11.89277	11.11754	9.64664	0.88402	1.02769	-0.77871
51	2.90010	4.53239	2.47290	0.93039	0.18458	0.12129
52	3.46136	3.03166	3.52277	-0.89713	1.05479	-1.26526
53	2.68893	2.15911	4.62362	-0.06346	-0.11285	0.11705

54	3.69069	4.90484	1.89536	0.63034	0.10273	0.41891
55	3.80600	2.32304	3.73815	0.15020	-0.59325	-0.31271
56	2.95467	2.35655	4.16507	0.08695	-0.28133	-0.42227
57	4.04614	4.29982	1.79834	-0.49439	0.74581	-0.54881
58	3.78339	2.12685	3.52779	0.56939	-0.23586	0.11038
59	3.82317	3.96707	2.56227	-1.19993	0.82770	-1.19803
60	5.13744	2.84152	2.46212	0.70654	-0.68335	-0.74529
61	2.97482	2.79389	3.81323	0.13856	0.05006	0.64532
62	3.80806	2.00349	3.79652	0.64590	-0.51363	-0.04269
63	4.17652	3.84112	2.14554	0.12756	1.12692	-0.46196
64	3.24710	4.32443	2.26954	-0.19232	0.55788	-0.93978
65	4.09186	1.98675	3.54286	0.05766	-0.55017	0.39814
66	4.79163	1.68203	3.57770	0.29905	-0.74438	-0.08049
67	3.88410	3.99267	2.24446	0.53434	0.65737	0.93992
68	4.26336	3.46236	2.43382	0.71737	1.18364	0.51190
69	4.05278	1.86131	3.61611	-0.08987	-0.70780	-0.04660
70	4.15522	2.20716	3.86379	0.30880	-1.11617	0.00008
71	3.40444	3.44016	2.81759	0.64822	1.00760	0.61704
72	3.73272	2.10323	3.79666	0.16167	-1.04834	-0.50994
73	3.82066	2.41614	3.76681	0.31849	-1.47657	-0.54030
74	2.36009	5.25645	2.18404	0.42442	0.12710	-0.83942
75	2.50719	4.48290	2.74807	0.77715	0.34778	-0.46402
76	4.18101	3.06167	2.73723	-1.44019	1.19324	-0.95300
77	3.20495	2.14665	4.41860	0.45271	-1.20718	-0.70222
78	4.52803	1.93900	4.00538	0.42280	-1.51280	-0.17873
79	2.75049	3.33618	3.62508	0.48567	0.70473	1.49432
80	2.58671	3.46324	3.49745	0.04363	0.40528	0.92001
81	5.41731	1.94481	2.95598	-0.22837	1.33688	-0.38828
82	3.24537	2.03942	4.60557	0.26922	-1.22898	-0.71414
83	3.76394	1.92902	4.09886	0.18689	-0.89200	0.11619
84	3.55975	3.50539	2.66738	0.16378	0.30461	-0.90422
85	3.53730	2.57727	3.89101	0.27683	-1.44386	-0.45744
86	5.43263	2.78594	1.87988	-0.41669	0.51385	0.00577
87	2.47293	4.05117	3.22933	0.47405	0.47690	1.55041
88	4.03935	2.71701	3.14472	0.10714	-0.36382	1.27904
89	4.09568	2.63540	3.11771	-0.10912	0.43901	-0.95047
90	3.24766	3.97477	2.65883	0.62025	-0.50915	-0.49492
91	5.68670	2.04547	2.50912	-0.39746	0.00846	-0.08070
92	3.86186	1.84642	4.56911	0.49526	-0.80665	-0.27131
93	3.28589	4.31605	2.10393	0.78093	-0.17810	0.21078
94	4.60153	2.10994	3.79572	0.79351	0.67574	0.63342
95	3.02396	4.30164	2.39257	0.36712	-0.27794	-0.77891
96	3.14733	2.53124	3.99150	0.02681	0.37955	-1.13925
97	5.53189	3.00477	1.86486	-0.31695	-0.16881	0.02180
98	3.98812	4.45610	1.90839	1.52684	-0.27218	-0.27150
99	3.43245	2.28770	3.94403	0.06110	0.34899	-0.23434
100	2.91125	2.85033	3.85502	0.57196	0.35266	-0.44463
101	5.77713	2.81041	1.90889	1.07485	-0.35658	0.36302
102	4.28604	3.50550	1.95210	-1.22372	-0.25031	0.23106
103	2.25268	2.48722	4.70958	0.07136	-0.07349	-0.14898
104	2.20466	4.18109	3.52801	0.25011	-0.02038	1.63565
105	5.00843	3.11583	2.08662	-0.99259	-0.04778	0.08492
106	3.71386	4.08164	2.04625	1.43773	-0.21794	-0.06478
107	4.21134	3.46369	2.07799	-1.43562	0.18865	-0.28542
108	5.31073	2.89491	2.03586	-1.27448	0.05117	-0.36318
109	4.26571	3.38544	2.45098	1.64601	0.22431	-0.03959
110	2.42349	3.12008	4.31253	0.17072	-0.29962	-1.51152
111	5.50499	3.53657	2.14036	1.52885	-0.99840	-0.54581
112	2.95607	4.56599	2.34611	-0.36296	-0.44443	0.75143
113	3.96451	2.47012	3.36494	-0.02507	1.05781	-0.40707

114	4.70226	2.36977	2.63545	-0.32718	0.39445	-0.54589
115	2.63643	4.54182	2.46315	-0.06675	-0.64914	0.38230
116	4.43613	2.27559	2.93894	0.25173	0.74691	-0.48828
117	4.01528	2.26826	3.22634	-0.00765	0.73008	-0.69156
118	2.75142	4.24508	2.72199	-0.28817	-0.89204	0.43006
119	2.53996	4.92255	2.28497	-0.24149	-0.39426	0.19320
120	5.01215	2.25514	2.59087	0.20450	0.60831	-0.34537
121	2.76034	2.29118	5.22353	0.44708	-1.04930	-0.45535
122	3.31884	2.27942	4.05982	-0.27816	-0.34133	0.91267
123	4.37444	3.63753	2.04554	1.01845	-0.15880	-0.57840
124	4.43550	3.74507	1.74368	0.62315	0.48409	-0.09722
125	4.02251	2.12811	3.48002	-0.59915	-0.01841	0.50476
126	3.58155	2.00710	4.10459	-0.46900	-0.41294	0.51233
127	4.05066	3.83699	1.92407	0.64649	0.68064	0.02559
128	3.19505	4.45744	2.05295	0.41676	0.55668	0.35109
129	3.93630	1.82813	3.76815	-0.30151	-0.38350	0.03735
130	3.76723	2.04430	3.92893	-0.10848	-0.50424	0.19312
131	3.67143	4.40878	2.03001	0.59336	0.77955	0.60281
132	2.72866	4.93181	2.17890	0.08612	0.40454	0.56364
133	3.47452	2.02872	4.01116	0.11411	-0.67219	-0.21367
134	3.76106	2.44935	3.45984	-0.29959	-1.02109	0.41921
135	2.93392	4.01371	3.12494	-0.71686	0.84540	-1.38619
136	4.35724	2.39996	3.44792	-0.22081	1.72752	-0.42252
137	3.77775	2.35210	3.43617	-0.12719	-0.81991	0.46235
138	4.93223	2.31755	2.52586	-0.15464	-0.86530	0.05663
139	2.69908	3.73190	3.47487	0.77149	0.74851	1.31570
140	4.72253	2.76232	2.16220	-0.41635	0.32587	-0.49037
141	2.38097	2.72465	4.68856	0.49180	-0.80125	-0.12394
142	5.47588	2.42186	2.13029	-0.58385	-0.04978	-0.34453
143	2.89236	4.68662	2.18157	0.67352	-0.36746	0.09247
144	5.39896	2.36366	2.31048	-0.27846	-0.13464	-0.15905
145	2.37585	3.67777	3.63593	-0.24589	-0.64154	1.23187
146	3.09827	3.53006	3.50124	0.95958	0.74270	0.95614
147	3.00290	4.62061	2.67651	1.27422	-0.40729	-1.24534
148	4.72894	2.77053	2.36947	-0.46493	-0.55775	0.38197
149	5.08045	2.87138	2.56404	-0.37494	0.43214	-0.86900
150	3.16532	2.23885	4.42762	-0.03975	-1.07698	0.45590
151	5.07803	3.29601	2.00023	-0.92512	0.25312	0.06239
152	3.58404	3.18079	3.30335	0.92472	0.56586	1.27473
